# What Do Studies of Insect Polyphenisms Tell Us about Nutritionally-Triggered Epigenomic Changes and Their Consequences?

**DOI:** 10.3390/nu7031787

**Published:** 2015-03-11

**Authors:** Andrew G. Cridge, Megan P. Leask, Elizabeth J. Duncan, Peter K. Dearden

**Affiliations:** Gravida and Genetics Otago, Biochemistry Department, University of Otago, P.O. Box 56, Dunedin 9016, New Zealand; E-Mails: andrew.cridge@otago.ac.nz (A.G.C.); megan.leask@otago.ac.nz (M.P.L.); elizabeth.duncan@otago.ac.nz (E.J.D.)

**Keywords:** polyphenisms, epigenetics, DNA methylation, chromatin structure, insect models

## Abstract

Many insects are capable of remarkable changes in biology and form in response to their environment or diet. The most extreme example of these are polyphenisms, which are when two or more different phenotypes are produced from a single genotype in response to the environment. Polyphenisms provide a fascinating opportunity to study how the environment affects an animal’s genome, and how this produces changes in form. Here we review the current state of knowledge of the molecular basis of polyphenisms and what can be learnt from them to understand how nutrition may influence our own genomes.

## 1. Introduction

Epigenetic mechanisms can be regulated or induced by the environment, and lead to long-term changes in gene expression, making them ideally suited to acting as mediators of diet-induced changes in phenotype. The problem is that they are complex, and the changes in phenotype they influence are often subtle or tissue specific. We need to understand how epigenetic mechanisms respond to their environment, how they are targeted to influence the expression of specific genes, and how these genes act to change phenotype. The complexity of these mechanisms and their outcomes makes understanding the fundamental biology involved difficult.

Insects provide an ideal model system to study the role of epigenetics in environmentally induced phenotypic change. Most insects methylate their DNA [1] as humans do. Insects, like all animals, have a conserved chromatin modification system similar to humans, and many of them respond, in a plastic and predictable way, to environmental cues. These environmental responses range from subtle colour variations and seasonal changes in form, to the most extreme changes; polyphenisms.

**Figure 1:**
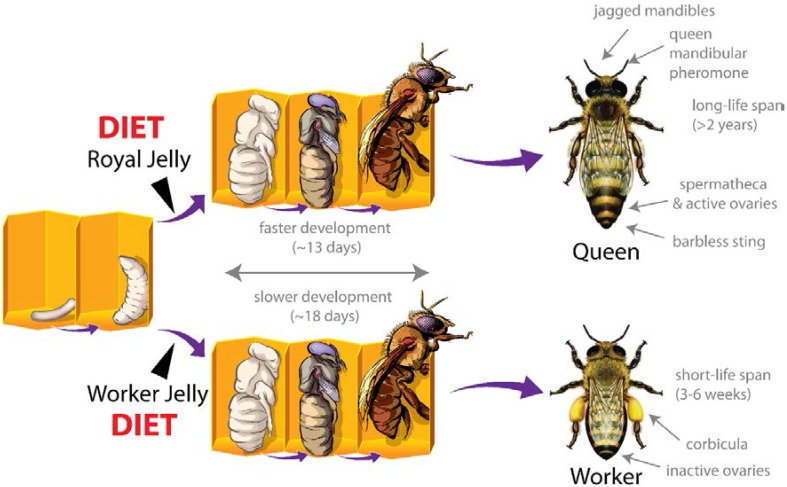
Honeybee biology depends on a polyphenism that produces different female castes. During larval development, female larvae fed royal jelly (top) develop faster and pupate earlier; producing queen bees. Female larvae fed worker jelly have slower development and produce worker bees. This diet-induced change in phenotype is robust and predictable and provides an opportunity to explore how diet affects the genome, and how this leads to changes in form.

Polyphenisms are where two or more distinct phenotypes are produced from a single genotype. Perhaps the best-known case of this is caste development in honeybees. Honeybees have two female castes, workers and queens. They differ in behaviour and physiology, not because of different genetics, but as a consequence their larval diet (Figure 1). Larvae destined to become queens are fed royal jelly (RJ), a nutrient rich diet, while worker larvae are fed a more dilute diet known as worker jelly [2]. This difference in larval nutrition sets up different developmental trajectories that are characterised by changes in gene expression throughout larval development [3]. Larval diet changes gene expression, which in turn influences female honeybees to become workers or queens (Figure 1). Polyphenisms are often associated with caste and are found in many insects (e.g., bees, ants, beetles), providing an opportunity to study the epigenetic mechanisms involved in systems where the environmental trigger is known and the phenotypic outcome extreme.

Understanding the role of epigenetic mechanisms in insect polyphenisms is perhaps the best way to begin to untangle the basic biology underpinning the impact of diet on phenotype. Such studies are beginning to provide important insights into nutritional epigenetics.

## 2. DNA Methylation and Nutritional Epigenetics

As stated above, honeybee caste development represents an important model system in which to study the impact of diet on the epigenome. DNA methylation, the reversible addition of a methyl group to a cytosine residue in the DNA, is one way in which larvae respond to differences in their nutrition. Reducing the expression of the enzyme that establishes DNA methylation marks *(Dnmt3*) by RNA interference results in a bias towards queen, rather than worker development [4]. If a bee is unable to make new DNA methylation marks during larval development, it develops as a queen bee. DNA methylation is thus required to make workers. This demonstrates a link between nutrition, differential DNA methylation, gene expression and phenotype in a way that has not been achieved in other animals, and raises the possibility that DNA methylation may underpin diet induced changes in phenotype. While we know that DNA methylation is important, we do not understand how this difference in nutrition alters DNA methylation, nor how the differences in DNA methylation are linked to changes in gene expression and ultimately the differences in adult phenotypes seen in queen and worker bees.

DNA methylation is a conserved mechanism found in all branches of animal life. DNA methylation has multiple functions; in mammals, DNA methylation in the promoter region of genes has been associated with imprinting and gene silencing [5], but in the majority of animals, including mammals, DNA methylation also occurs on gene bodies (transcription units) [6], as it does in honeybees [7]. Gene body methylation is associated with diverse functions such as alternative splicing [8,9,10], repressing intragenic promoter activity [11] and controlling transcriptional elongation [12]. It is suggested that DNA methylation acts with other epigenetic mechanisms, such as histone modifications and non-coding RNAs, to regulate gene expression in mammals [13] and insects [14].

DNA methylation is important for regulating how honeybee larvae respond to nutrition during development [4]; but how does nutrition alter DNA methylation? Do components of our diet directly influence DNA methylation, or is the effect on DNA methylation indirect? In other experimental models altering the pool of methyl-donors in the diet influences DNA methylation [15,16,17]. This does not explain the effect of RJ on DNA methylation, as the major biological activity of RJ has been reported to be associated with a protein called royalactin, rather than micronutrients or methyl-donors [18]. Royalactin has been linked to alterations in epidermal growth factor (EGF) signalling [18], which influences DNA methylation in mammals [19,20], but a direct link between EGF signalling and DNA methylation has not been shown in honeybees. It seems likely that the influence of RJ on DNA methylation is indirect, and mediated by cell signalling systems.

Changes in DNA methylation have been linked to larval nutrition in a beetle [21] and caste specification in ants [22]. DNA methylation has also been linked with other, non-nutritional, polyphenisms in insects (*i.e.*, Phase transitions between solitary and gregarious locusts [23]), raising the possibility that it may be a molecular mechanism commonly used to mediate cellular responses to the environment.

In other animals, including mammals, diet is known to influence DNA methylation in individuals [24] and nutrition during pregnancy has been associated with differences in DNA methylation in tissues in the offspring [25]. Perhaps surprisingly, the diet of the father prior to conception alters DNA methylation in their offspring [26,27,28,29]. These studies imply that nutritional stimuli may target specific genes or regions of the DNA and that the regions targeted may depend on the exact nature of the nutritional stimulus. We do not currently have any mechanism to explain how such specificity might be regulated [5].

Studies in insects and mammals have shown that nutrition can have a profound effect on the phenotype of the animal and that, in some cases, these effects are mediated through DNA methylation. Yet the only system that this has been shown conclusively is caste development in the honeybee, where functional manipulation of the DNA methylation system has conclusively linked the nutritional intake with DNA methylation and adult phenotype [4]. This is a critical demonstration of how diet affects phenotypic change through epigenetic mechanism. It remains to be determined if DNA methylation is a general mechanism in all animals for environmental and nutritional response.

## 3. Histone Modifications and Insect Polyphenisms

Chromatin remodelling proteins, and the histone modifications they create, are highly conserved, and achieve broad regulation of gene expression by influencing the three dimensional structure of chromatin, and the recruitment of transcription factors to DNA [30,31]. This form of epigenetic regulation of gene expression is essential for the correct establishment of developmental programs and for the maintenance of cell fates [32,33]. In insect polyphenisms, large-scale gene expression differences are a hallmark of the alternative phenotypes produced by environmental exposure [3,34]. Is chromatin remodelling responsible for the establishment of these alternative developmental programmes and their maintenance? If so, how do nutritional stimuli bring about changes in chromatin structure?

In an ant, *Harpegnathos saltator*, several members of the SET and MYND domain-containing protein (SMYD) family of histone methyltransferases, have been identified as differentially expressed between castes [35]. In addition expression analyses in honeybee have shown that chromatin remodelling genes also have differential expression between alternative castes [36]. The most convincing evidence that chromatin modifications have a role in nutritionally induced polyphenism is from studies of the Carpenter ant (*Camponotus floridanus*). Chromatin immunoprecipitation followed by sequencing (ChIP-seq), indicates that differences in chromatin structure near protein coding genes occur between the two types of worker castes (minors and majors). In particular, it was found that acetylation of histone H3 at lysine 27 (H3K27ac), a modification generally considered to activate transcription, could predict caste type, and that CREB-binding protein, the enzyme responsible for this modification, is expressed in a caste-specific manner [37]. This finding may also provide a better understanding of the activity of RJ on honeybees as RJ contains a histone deacetylase inhibitor [38], which may regulate the distribution or amount of H3K27ac in key tissues of the larva and thereby facilitate its development into a queen. At least one histone deacetylase, Sirtuin 2 (Sir2), is expressed at higher levels in the heads of adult queen bees compared with non-reproductive worker bees [36]. It is unknown if the differences in Sir2 RNA cause functional changes in histone acetylation and whether these differences originate as a result of the different nutritional stimuli during larval development or whether they are established later in life as a downstream or secondary effect of this stimulus. Sir2 is also nutritionally responsive in honeybees, with a low protein diet associated with an increase in global levels of Sir2 RNA and an extension of lifespan [39]. The induction of Sir2 by a low-protein diet is intriguing as it may give us clues as to how queen bees have an extended life span (>2 years) compared with worker bees (3–6 weeks).

While these studies indicate that chromatin modifiers and histone modifications change in response to nutritionally induced polyphenisms, it is unclear if this is a cause or an effect. Does chromatin remodelling play a functional role in the establishment or maintenance of nutritional polyphenisms? Or does it merely respond to a changing transcriptional landscape perhaps triggered by DNA methylation or transcription factor activity? [14]

## 4. Other Epigenetic Mechanisms and Insect Polyphenisms

A number of studies in bees and ants investigating dietary induced polyphenisms have proposed that post-transcriptional regulation of mRNA expression and translation plays a role in the links between diet and establishment of different developmental trajectories.

### 4.1. Alternative Splicing

The spliceosome is assembled from snRNPs and protein complexes in the nucleus of eukaryotic cells and removes introns from transcribed pre-mRNAs giving rise to alternative splice variants. Spliceosome encoding genes are differentially methylated between *Apis mellifera* castes [8], and a link has been proposed between gene-body methylation and control of alternative splicing in the honeybee [7,8,10,40] and the ant [22]. A functional link between DNA methylation and alternative splicing in the honeybee has recently been established by reducing the expression of the *de novo* DNA methyltransferase (*dnmt3*) in adult bees and demonstrating an effect on mRNA splicing, in particular exon skipping and intron retention [41]. In *A. mellifera,* queen larvae have consistently higher expression of genes associated with the spliceosome compared to worker bees. This correlates with the finding that ~50% of genes expressed in bee larvae have alternative transcripts. Both queens and worker larvae have greater expression of multiple splice variant genes [3]. However, both queens and workers tend to show higher expression of a single variant rather than multiple variants of the same gene. In the majority of cases a single transcript is differentially regulated, while the other transcript(s) for these genes remain constitutively expressed [3]. Thus evidence for alternative splicing contributing to the queen and worker developmental trajectories is inconclusive.

### 4.2. RNA-Editing

Post-transcriptional gene regulation via RNA editing alters RNA sequences through individual base substitutions, insertions or deletions [42,43]. The most prevalent type of RNA editing in the animal kingdom is A-to-I editing, where adenosine (A) residues are converted to inosine (I) catalysed by adenosine deaminase (ADAR) enzymes that use double-stranded RNAs (dsRNAs) as substrate [43]. As inosine is recognized as guanosine by the ribosome during translation, A-to-I editing in protein-coding sequences may result in amino acid changes that alter the functional properties of proteins [43]. A-to-I editing can also play an important role in regulating gene expression by affecting alternative splicing [44], editing microRNA (miRNA) sequences [45] or changing miRNA target sites in messenger RNA [46]. Recent studies show that RNA editing might enhance the diversity of gene products at the post-transcriptional level, particularly to induce functional changes in the development of specific castes of the leaf-cutting ant *Acromyrmex echinatior* [47].

### 4.3. Gene Regulation by Non-Coding RNAs

Various types of ncRNAs, including microRNAs (miRNAs), PIWI-interacting RNAs (piRNA) and long ncRNAs, can regulate development, and thus potentially polyphenisms through transcriptional and post-transcriptional gene regulation [48,49]. Conventionally, ncRNAs are divided into short (<200 nucleotides) and long (>200 nucleotides) classes.

### 4.4. Short ncRNA

Both conserved and lineage-specific short ncRNAs, including miRNAs and piRNA, have been identified in ants and honeybees, and many of these short ncRNAs show caste-specific expression [37,50].

### 4.5. miRNA

miRNA are small ncRNA molecules (containing about 22 nucleotides) found in plants, animals, and some viruses, which functions in RNA silencing and post-transcriptional regulation of gene expression. Investigations into the composition of larval bee food have identified that worker jelly is enriched in miRNA complexity and abundance relative to RJ [51,52]. miRNA levels in worker jelly were 7–215 fold higher than in RJ, and both jellies showed dynamic changes in miRNA content during the 4th to 6th day of larval development. Adding the miRNA miR-184, which is more abundant in worker jelly, to RJ elicited significant changes in queen larval mRNA expression and morphological characters of the emerging adult queen bee [51]. These findings imply that miRNAs are an additional element in the regulatory control of caste determination in the honeybee.

### 4.6. PIWI RNA

PIWI genes are expressed in the germ-line and play an important role in regulating spermatogenesis and oogenesis through regulating germ-line determination and germ-line stem cell maintenance to meiosis, spermatogenesis, and transposon silencing. PIWI proteins participate in the biogenesis of a novel class of small RNAs known as PIWI-interacting RNAs (piRNAs). Most piRNAs are generated from long single-stranded RNA precursors often encoded by repetitive intergenic sequences in the genome. The diverse functions of piRNAs may be achieved via novel mechanisms of epigenetic and post-transcriptional regulation. Honeybees contain two genes encoding PIWI-like proteins, *Am-aub* and *Am-ago3*. Both *Am-aub* and *Am-ago3* are more abundant in queens than workers, indicating that larval food influences the long-term expression of PIWI genes. The genes coding for small non-coding RNAs, known as piRNAs, are differentially methylated in queens and workers of the black garden ant (*Lasius niger*). These piRNAs are instrumental for targeting DNA methylation to transposable elements, especially in germ cells during developmental re-programming, and are thus a key factor in granting germline cells infinite numbers of division. [53]. How nutritional stimuli bring about changes in piRNA expression and if the changes in piRNA levels is a direct response of nutrition or a secondary function of queen development is unknown [35].

### 4.7. lnRNA (Long Noncodingrna)

Long ncRNAs share many characteristics with mRNAs, such as a multiexonic structure, polyadenylation and transcription by RNA polymerase II (Pol II); many long ncRNAs also have tissue-specific expression patterns [54]. As several long ncRNAs can interact with both genomic DNA and epigenetic regulators, they may recruit or stabilize epigenetic modifications at specific genomic loci in a similar manner to transcription factors (although probably in *cis*) [48]. Although there has not yet been a comprehensive identification of long ncRNAs in any eusocial insect, two long ncRNAs were recently characterized in the honeybee in association with regulation of worker ovary size [55]. Expression analysis on larval worker ovaries indicated an expression peak of these lcRNA coinciding with the onset of autophagic cell death, which is key to worker, but not queen, development.

## 5. Conclusions

Epigenetic mechanisms are complex and subtle, and we need simple experimental systems to understand their role in responding to our diet. While insects are not simple, insect polyphenisms present excellent experimental systems to probe the basic biology of epigenetic response to diet. That we can manipulate the environment, track related epigenetic changes, and see how those epigenetic effects influence the whole phenotype, and perhaps fitness of the insect, make these studies unique in their scope. Studies of polyphenisms in bees and ants, in particular, have provided important insights into the roles of DNA methylation and histone modifications that inform our understanding of these processes in human nutrition. The next challenges are to track down the critical epigenetic changes mediating environmental influence to the cells types in which they have their effect. Only by using single cell techniques, and then building our understanding of the whole phenotype from changes in the activity of those cells, will we gain a complete molecular and cellular understanding of polyphenisms. Further studies of this kind focusing on the role of epigenetics in insect polyphenisms may provide the key basic biology that unlocks our understanding of, and intervention in, diet induced human conditions.
